# Biomechanical analysis of a newly developed interspinous process device conjunction with interbody cage based on a finite element model

**DOI:** 10.1371/journal.pone.0243771

**Published:** 2020-12-11

**Authors:** In-Suk Bae, Koang-Hum Bak, Hyoung-Joon Chun, Je Il Ryu, Sung-Jae Park, Sung-Jae Lee

**Affiliations:** 1 Department of Neurosurgery, Eulji University Eulji Hospital, Nowon-gu, Republic of Korea; 2 Department of Neurosurgery, Hanyang University Medical Center, Seongdong-gu, Seoul, Republic of Korea; 3 Department of Neurosurgery, Hanyang University Guri Hospital, Guri, Gyonggi-do, Republic of Korea; 4 R&D Center, GS medical Co, Ltd, Cheongju-si, Chungcheongbuk-do, Republic of Korea; 5 Department of Biomedical Engineering, College of Biomedical Science& Engineering, Inje University, Gimhae-si, Gyeongsangnam-do, Republic of Korea; Assiut University Faculty of Medicine, EGYPT

## Abstract

**Purpose:**

This study aimed to investigate the biomechanical effects of a newly developed interspinous process device (IPD), called TAU. This device was compared with another IPD (SPIRE) and the pedicle screw fixation (PSF) technique at the surgical and adjacent levels of the lumbar spine.

**Materials and methods:**

A three-dimensional finite element model analysis of the L1-S1 segments was performed to assess the biomechanical effects of the proposed IPD combined with an interbody cage. Three surgical models—two IPD models (TAU and SPIRE) and one PSF model—were developed. The biomechanical effects, such as range of motion (ROM), intradiscal pressure (IDP), disc stress, and facet loads during extension were analyzed at surgical (L3-L4) and adjacent levels (L2-L3 and L4-L5). The study analyzed biomechanical parameters assuming that the implants were perfectly fused with the lumbar spine.

**Results:**

The TAU model resulted in a 45%, 49%, 65%, and 51% decrease in the ROM at the surgical level in flexion, extension, lateral bending, and axial rotation, respectively, when compared to the intact model. Compared to the SPIRE model, TAU demonstrated advantages in stabilizing the surgical level, in all directions. In addition, the TAU model increased IDP at the L2-L3 and L4-L5 levels by 118.0% and 78.5% in flexion, 92.6% and 65.5% in extension, 84.4% and 82.3% in lateral bending, and 125.8% and 218.8% in axial rotation, respectively. Further, the TAU model exhibited less compensation at adjacent levels than the PSF model in terms of ROM, IDP, disc stress, and facet loads, which may lower the incidence of the adjacent segment disease (ASD).

**Conclusion:**

The TAU model demonstrated more stabilization at the surgical level than SPIRE but less stabilization than the PSF model. Further, the TAU model demonstrated less compensation at adjacent levels than the PSF model, which may lower the incidence of ASD in the long term. The TAU device can be used as an alternative system for treating degenerative lumbar disease while maintaining the physiological properties of the lumbar spine and minimizing the degeneration of adjacent segments.

## Introduction

Spinal fusion is a common procedure for the surgical treatment of lumbar degenerative diseases [1, 2]. Spinal fusion using pedicle screw fixation (PSF) technique combined with posterior lumbar interbody fusion has several advantages such as high rate of fusion, increase the height of intervertebral disc space, and maintaining the stability of spine [3, 4]. Although the PSF technique is a popular surgical method for the treatment of lumbar degenerative disease, it is technically demanding and can result in several complications [5]. In addition, the rigid fixation of lumbar spine can increase the range of motion (ROM) and intradiscal pressure (IDP) of adjacent levels, and cause adjacent segment diseases (ASDs) [6].

Alternative techniques were developed to overcome the disadvantages associated with the PSF technique. Interspinous process devices (IPDs) are widely used for the treatment of lumbar degenerative diseases. IPDs aim to provide stabilization after decompression surgery, restore the height of intervertebral foramen, and reduce the loads at the facet joints [7]. Implantation of IPD has been reported in several studies to be effective in the surgical treatment of degenerative spinal diseases [7–9].

Recently, a newly developed IPD, TAU (GS Medical, Korea), has been introduced. This device is an alternative to traditional PSF systems, and used as an adjuvant to an interbody cage in spinal fusion. In this FE study, the biomedical effects on the lumbar spine using the newly developed IPD were compared with that of another IPD (SPIRE, Medtronic Sofamor Danek, Memphis, TN, USA) and the PSF (Anyplus screw, GS Medical Co., Ltd., Korea) system by using a hybrid testing protocol.

## Materials and methods

### Study design

**Fig 1:**
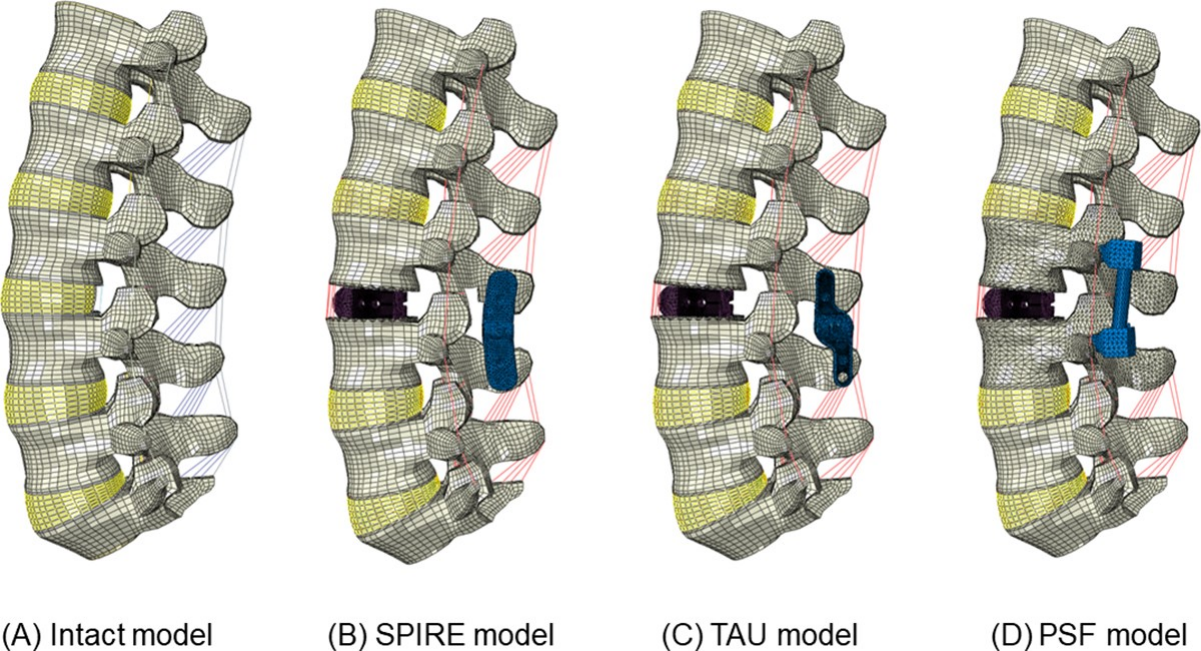
Finite element model of the intact and implanted lumbar spine: (A) Intact model, (B) SPIRE model with interbody cage, (C) TAU model with interbody cage, (D) Pedicle screw fixation model with interbody cage.

A previously validated, 3-dimensional, intact, osteoligamentous lumbar spinal segment model of L1-S1 was used [10]. The geometry of the lumbar spine vertebrae was obtained from computed tomography (CT) data of a healthy 44-year-old male. The material properties of each component were obtained from the literature, as summarized in Table 1 [11–13]. The final FE model had six vertebrae (L1-S1), five intervertebral discs, cartilage endplates, and ligaments. The total number of nodes and elements were 69,337 and 68,816, respectively. The FE model was exported to ABAQUS software (ABAQUS 6.13; Hibbitt, Karlsson & Sorenson, Inc., Providence, RI, USA) after the material properties were applied to each component of the lumbar spine model. A total of four FE models of the lumbar spine were constructed in this study. The first one was a model of a healthy lumbar spine. The other three models were the implanted models, where two were implanted with IPDs (TAU and SPIRE) and the other used a PSF system (Fig 1).

**Table 1:** Material properties used in the finite element models.

Materials	Young’s modulus (MPa)	Poisson’s ratio	Cross-sectional area (mm^2^)
**Bony structure**			
Cortical bone	12,000	0.3	
Cancellous bone	100	0.2	
Posterior element	3,500	0.25	
End plate	25	0.25	
Annulus ground	4.2	0.45	
Nucleus pulposus	1.0	0.499	
**Annulus fibers**			
Layer 1, 2	550		0.5
Layer 3, 4	495		0.39
Layer 5, 6	413		0.31
Layer 7, 8	358		0.24
**Ligaments**			
ALL	7.8 (< 12%), 20 (> 12%)		63.7
PLL	10 (< 11%), 20 (> 11%)		20
LF	15 (< 6.2%), 19 (> 6.2%)		40
CL	7.5 (< 25%), 33 (> 25%)		30
ITL	10 (< 18%), 59 (> 18%)		1.8
ISL	10 (< 14%), 12 (> 14%)		40
SSL	8 (< 20%), 15 (> 20%)		30

ALL: anterior longitudinal ligament; PLL: posterior longitudinal ligament; LF: ligament flavum; CL: capsule ligament; ITL: intertransverse ligament; ISL: interspinous ligament; SSL: supraspinous ligament.

### FE model of interspinous process devices and PSF technique (implanted model)

To compare the biomechanical changes after lumbar spine surgery, three types of surgical models -two IPD models and one PSF model- were developed based on the validated intact model. The geometries of the implants were recreated by PTC Creo Parametric 4.0 software (Parametric Technologies Corp., MA, USA) from the real product and then transferred into the ABAQUS software to construct the FE model.

The PSF model consisted of two interbody cages, four pedicle screws, and two rods. The material used for the pedicle screws and rods was Ti-6Al-4V alloy. Young’s modulus and Poisson’s ratio were assigned to be 114,000 MPa and 0.3, respectively. The length and diameter of the pedicle screw were 45 mm and 6 mm, respectively. Further, the length and diameter of the rod were 80 mm and 6 mm, respectively. The pedicle screws were simplified as cylinders. Following the standard surgical method, the pedicle screw placement was performed using Weinstein’s method [14], and screws were inserted parallel to the superior endplate. Then, the interbody cage was inserted at the disc space between L3 and L4. The material of the cage was polyetheretherketone; moreover, the Young’s modulus and Poisson’s ratio of the cage were assigned to be 3,600 MPa and 0.3, respectively. A “Tie” interaction was applied to the interbody cage and vertebral bodies for complete fusion.

The IPD model consisted of two interbody cages and the IPD. The interbody cages were inserted at the same place as in the PSF model. The IPD (TAU and SPIRE) device is comprised of two titanium plates with aggressive, opposing spikes connected at their midpoint. The surgical technique of IPD was performed as described by Kim et al. [15]. To implant the IPDs, a part of the L3-L4 interspinous ligament was removed to obtain congruent contact surfaces [16]. IPD was inserted between the cranial and caudal portions of the spinous processes, and each blade ensures maximal contact with its respective spinous process [17]. Also, the “Tie” interaction was applied to the IPD and spinous process for complete fusion. The IPDs were constructed using Ti-6Al-4V alloy. The Young’s modulus and Poisson’s ratio were assigned to be 113,000 MPa and 0.3, respectively. All models were verified by an experienced surgeon.

### Boundary and loading condition

Two loading conditions were used in this study as described by Choi et al. [18]. The first loading condition was for validating the intact FE model, following the same protocol used in the study by Yamamoto et al. [19]. The second loading condition was applied to both the intact and surgical models to analyze the biomechanical changes after surgery caused by the PSF or IPD. A follower load of 400 N, which represents a partial body weight along the curvature of the lumbar spine, was applied [20]. The truss elements were attached bilaterally along the curvature of the lumbar spine, as discussed in previous studies [21]. The validation of the intact FE model and the biomechanical changes caused by the surgical implants were investigated in flexion, extension, lateral bending, and axial rotation. A hybrid loading protocol, was implemented to investigate biomechanical changes at adjacent segment by varying the moment until the overall deflection of the implanted models equaled the predicted deflection for the intact model [22, 23]. The data were normalized according to the intact model as percentage values under each loading condition.

## Results

### Range of motion

**Fig 2:**
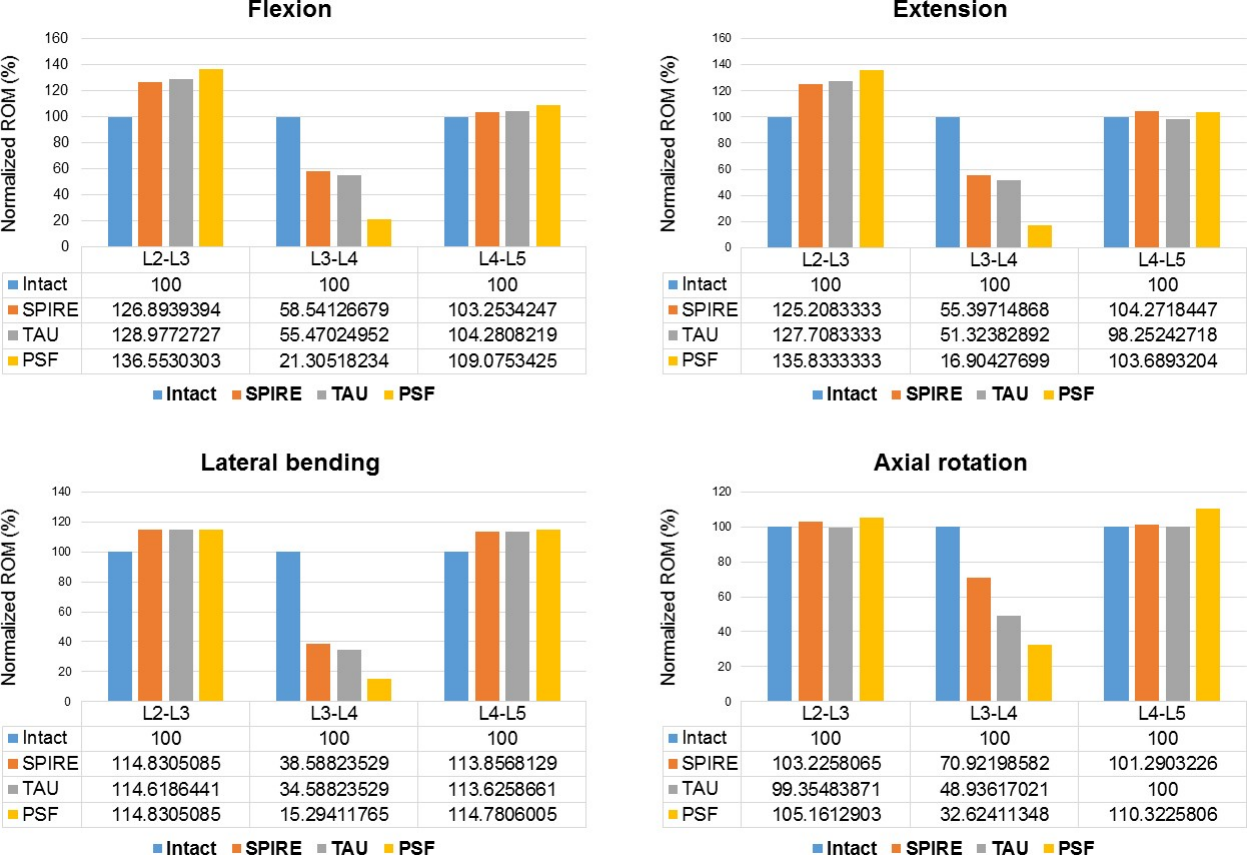
Range of motion for the intact and implanted lumbar models (Data were normalized with respect to the intact model as percentage values).

The results of the ROM are shown in Fig 2 and Table 2. The PSF model demonstrated a 79%, 83%, 85%, and 67% decrease in the ROM at the surgical level (L3-L4) in flexion, extension, lateral bending, and axial rotation, respectively, when compared to the intact model. The SPIRE model demonstrated a 41%, 45%, 61%, and 29% decrease in the ROM at the surgical level (L3-L4) in flexion, extension, lateral bending, and axial rotation, respectively, when compared to the intact model. Meanwhile, the TAU model resulted in a 45%, 49%, 65%, and 51% decrease in the ROM at the surgical level in flexion, extension, lateral bending, and axial rotation, respectively, when compared to the intact model. The TAU model revealed a greater decrease in the ROM at the surgical level in all directions when compared to the SPIRE model. However, the TAU model showed lesser decrease in the ROM at the surgical level in all directions when compared to the PSF model.

**Table 2:** Biomechanical parameters among various surgical devices (Data were normalized with respect to the intact model as percentage values).

		L2-3	L3-4 (surgical level)	L4-5
**ROM**				
Flexion	SPIRE	126.9	58.5	103.3
	TAU	129.0	55.5	104.3
	PSF	136.6	21.3	109.1
Extension	SPIRE	125.2	55.4	104.3
	TAU	127.7	51.3	98.3
	PSF	135.8	16.9	103.7
Lateral bending	SPIRE	114.8	38.6	113.9
	TAU	114.6	34.6	113.6
	PSF	114.8	15.3	114.8
Axial rotation	SPIRE	103.2	70.9	101.3
	TAU	99.4	48.9	100
	PSF	105.2	32.6	110.3
**IDP**				
Flexion	SPIRE	214.2		179.8
	TAU	218.0		178.5
	PSF	218.7		181.5
Extension	SPIRE	187.6		167.6
	TAU	192.6		165.5
	PSF	197.2		169.2
Lateral bending	SPIRE	182.3		185.9
	TAU	184.4		182.3
	PSF	184.8		186.5
Axial rotation	SPIRE	224.4		327.2
	TAU	225.8		318.8
	PSF	226.1		329.9
**Facet loads**				
	SPIRE	154.3	81.4	183.7
	TAU	154.6	76.0	188.4
	PSF	160.1	41.0	297.2

ROM: range of motion; PSF: pedicle screw fixation; IDP: intradiscal pressure.

At adjacent levels (L2-L3 and L4-L5), a relatively larger ROM was observed in the PSF model than in the IPD models (Fig 2). When compared to the intact model, the ROM for the PSF model corresponding to the L2-L3 and L4-L5 levels increased by 36.6% and 9.1% in flexion, 35.8% and 3.7% in extension, 14.8% and 14.8% in lateral bending, and 5.2% and 10.3% in axial rotation, respectively. For the SPIRE model, the ROM at the L2-L3 and L4-L5 levels increased by 26.9% and 3.3% in flexion, 25.2% and 4.3% in extension, 14.8% and 13.9% in lateral bending, and 3.2% and 1.3% in axial rotation, respectively. In addition, the ROM for the TAU model at the L2-L3 and L4-L5 levels increased by 29.0% and 4.3% in flexion, increased by 27.7% and decreased by 1.7% in extension, increased by 14.6% and 13.6% in lateral bending, respectively, and decreased by 0.6% in axial rotation (at L4-L5 level). The PSF model showed a greater increase in ROM at adjacent levels than IPD models. Further, the TAU model induced a minimum increase in ROM at adjacent levels in lateral bending and axial rotation among all the surgical models.

### Intradiscal pressure and disc stress at adjacent level discs

**Fig 3:**
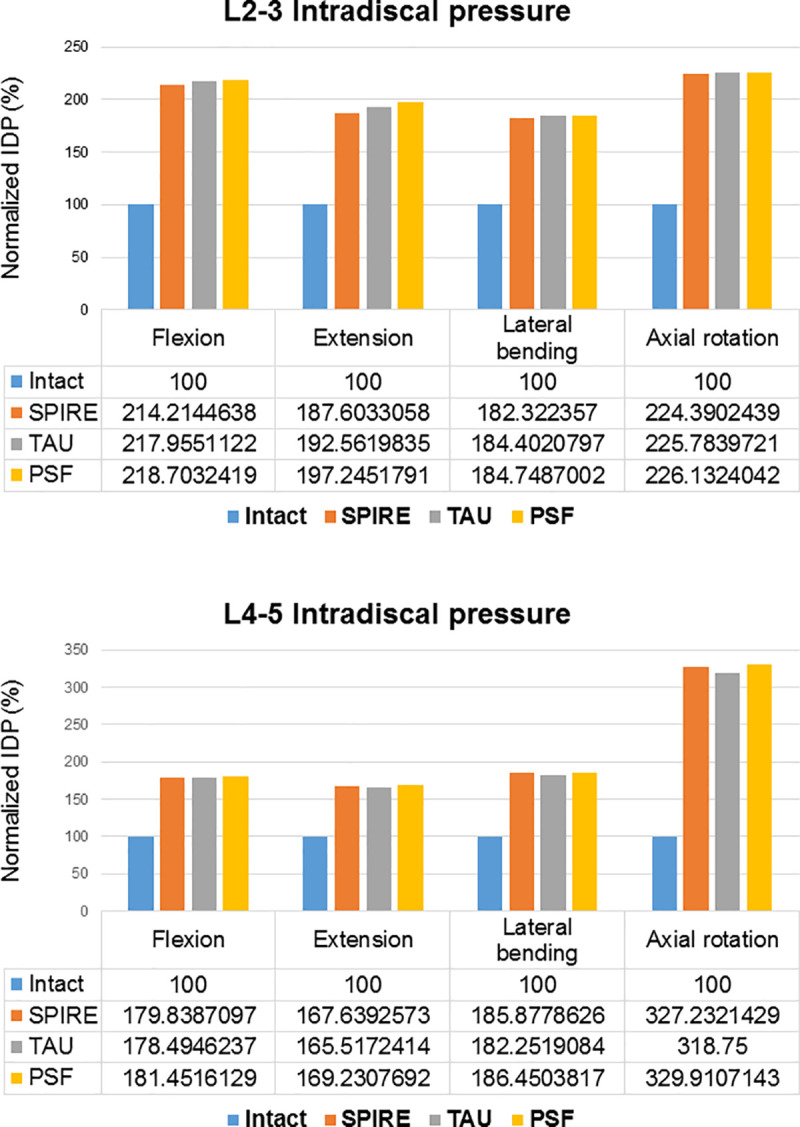
Intradiscal pressure at adjacent levels in implanted lumbar models (Data were normalized with respect to the intact model as percentage values).

The IDP at adjacent levels is displayed in Fig 3. All surgical models increased the IDP at the adjacent level substantially in flexion, extension, lateral bending, and axial rotation. When compared to the intact model, the PSF model increased IDP at the L2-L3 and L4-L5 levels by 118.7% and 81.5% in flexion, 97.2% and 69.2% in extension, 84.7% and 86.5% in lateral bending, and 126.1% and 230.0% in axial rotation, respectively. When compared to the intact model, the SPIRE model increased IDP at the L2-L3 and L4-L5 levels by 114.2% and 80.0% in flexion, 87.6% and 67.6% in extension, 82.3% and 85.9% in lateral bending, and 124.4% and 227.2% in axial rotation, respectively. The TAU model increased IDP at the L2-L3 and L4-L5 levels by 118.0% and 78.5% in flexion, 92.6% and 65.5% in extension, 84.4% and 82.3% in lateral bending, and 125.8% and 218.8% in axial rotation, respectively. The TAU model demonstrated a greater increase in IDP at superior adjacent level than the SPIRE model. However, the TAU model induced a minimum increase in IDP at the inferior adjacent level among all the surgical models. In addition, the PSF model showed a greater increase in the IDP than the IPDs (TAU and SPIRE) in all directions.

**Fig 4:**
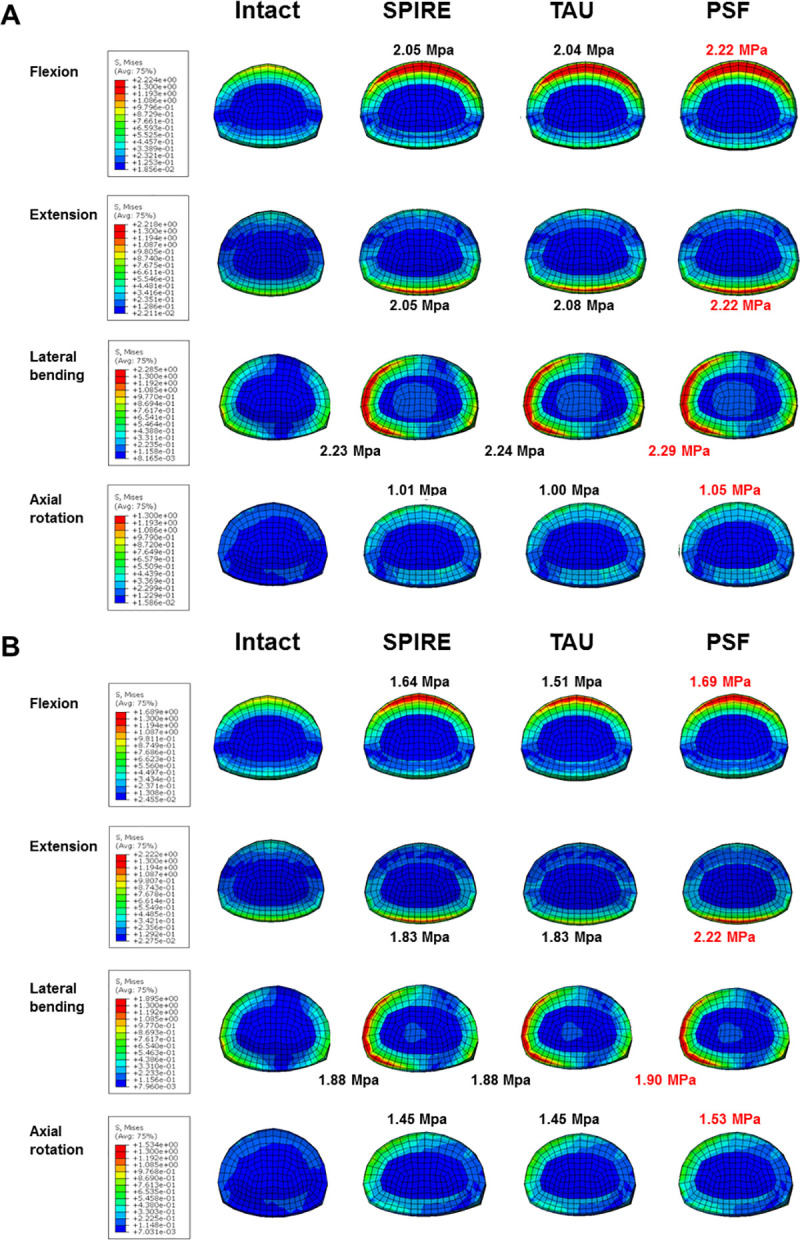
Stress distribution at the (A) superior (L2-L3) and (B) inferior (L4-L5) adjacent level discs.

The PSF system sustained the most disc stress at adjacent segments in all directions. The TAU model sustained lesser disc stress than the SPIRE model during flexion, but marginally more stress during extension. Meanwhile, the TAU model sustained almost the same disc stress as the SPIRE model during lateral bending and axial rotation (Fig 4). Further, the TAU and SPIRE models were observed to have similar disc stress distribution when compared to the intact model.

### Facet loads

**Fig 5:**
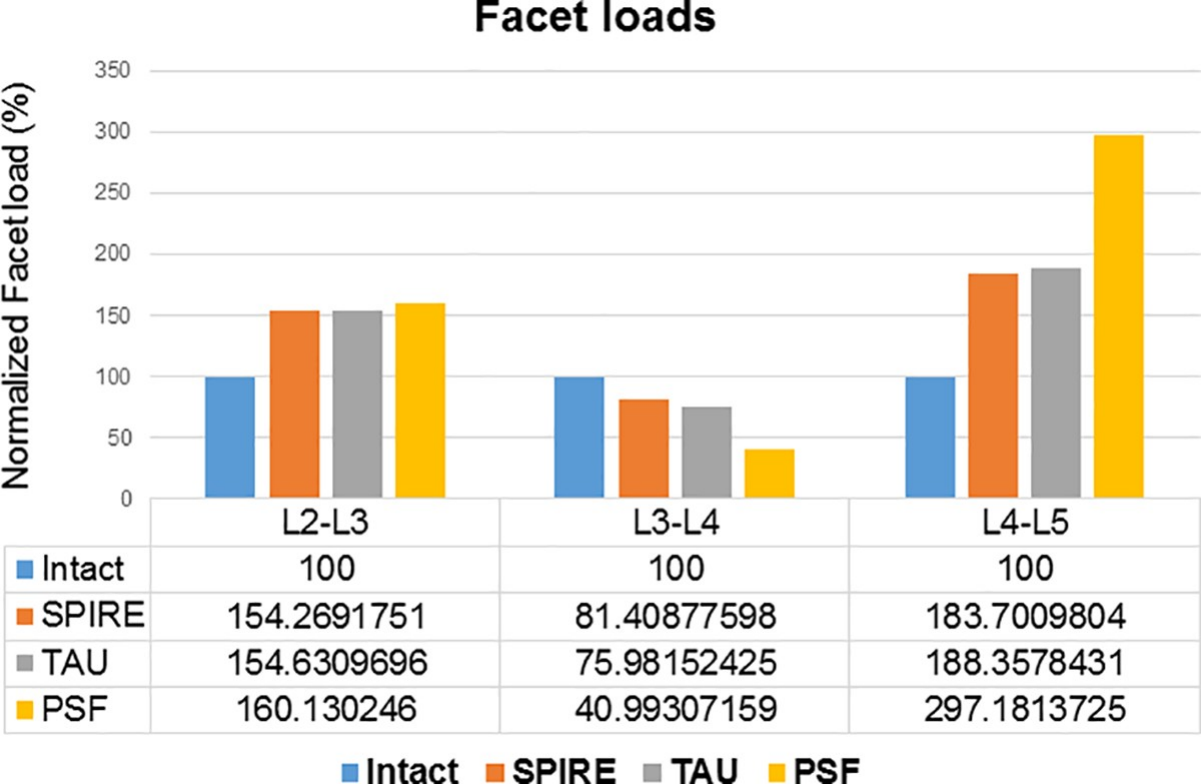
Facet loads during extension at surgical and adjacent levels in implanted lumbar models (Data were normalized with respect to the intact model as percentage values).

Fig 5 shows the facet loads at the surgical and adjacent levels. A comparison of the facet loads at the surgical and adjacent levels reveals that at the surgical level in the surgical model, the facet loads decreased significantly relative to the intact model. However, at the superior (L2-L3) and inferior (L4-L5) adjacent levels, the facet loads increased. When compared to the intact model, the facet loads at the superior level increased by 54.3% in the SPIRE model, 54.6% in the TAU model, and 60.1% in the PSF model. Further, the facet loads at the inferior level increased by 83.7% in the SPIRE model, 88.4% in the TAU model, and 197.2% in the PSF model. The TAU model showed lesser loads at the surgical level than the SPIRE model, while it showed marginally more loads at the adjacent levels than the SPIRE model.

## Discussion

In this study, the biomechanical changes after implantation of the new IPD- the TAU device- were investigated and compared with another IPD (SPIRE) and a PSF technique, using FE analysis. Recently, a newly developed IPD, TAU, was introduced; however, there is no biomechanical study regarding this new device. As suggested by Panjabi et al., a hybrid protocol presented the postoperative changes after lumbar spine surgery; moreover, it is appropriate for the biomechanical evaluation of adjacent spinal levels after spine surgery [22, 23]. Therefore, the used FE model was concluded to be appropriate for representing the surgical model of the lumbar degenerative disease and for evaluating the adjacent segment degeneration.

As expected, at the surgical level (L3-L4), the ROM in all surgical models was significantly decreased in all directions. In addition, the TAU model showed a greater stabilization effect at the surgical level than the SPIRE model. The FE analysis showed that the SPIRE and PSF models increased the ROM at adjacent levels, in all directions. However, the TAU model decreased the ROM at extension and axial rotation. Furthermore, the TAU model demonstrated a minimal change in ROM at adjacent levels in lateral bending and axial rotation.

IPDs are used with an interbody cage in lumbar spinal fusion surgery and can be used as an alternative to the PSF system [24]. The SPIRE device alone can provide immediate rigid fixation of the destabilized lumbar spine and to reduce ROM [17, 25]. In biomechanical studies comparing IPDs and PSF, IPDs reduced the ROM at flexion and extension to the same degree as PSF systems [17, 26]. Consequently, the TAU model showed comparable biomechanical effects with the PSF model. At the surgical level (L3-L4), the ROM in all directions was remarkably decreased. Conversely, at the adjacent level, the ROM, IDP, disc stress, and facet loads of the PSF model were remarkably increased according to the fusion effect. These adjacent effects were previously reported and considered as major complications of spinal fusion [21, 27].

The IDP at adjacent levels was directly affected by different devices. The PSF model significantly increased the IDP at adjacent levels. When compared to the PSF model, the TAU and SPIRE models showed lesser increase in IDP at adjacent levels. Furthermore, the IDP at adjacent levels was not significantly increased by the IPDs, which may be beneficial in preventing ASD after fusion surgery in the long term.

The disc stress after lumbar spine surgery at the adjacent level should not excessively exceed that of the intact model because excessive increase in the disc stress may accelerate the degeneration of adjacent segments. The TAU model marginally increased the disc stress at the adjacent level in all directions; further, it demonstrated almost the same results as the SPIRE model. However, the disc stress at the TAU model was significantly lower than that in the PSF model. These results reveal that the TAU device had minimal effect on disc stresses at adjacent levels, which is similar to the previous study [28]. Further, these results show that the IPDs may cause less degeneration at the adjacent levels than the PSF system.

In this study, the facet loads at adjacent levels of the TAU model increased marginally more than that of the SPIRE model during extension motion. The aim of the IPDs is to alleviate facet joint pain; therefore, surgeons should consider the facet loads at surgical and adjacent levels [29]. However, the facet loads at adjacent levels of the TAU model were lower than that of the PSF model. In addition, the facet loads at the surgical level of the TAU model were lower than that in the SPIRE model. These results indicate that when compared to the PSF model, the TAU model demonstrates advantages in decreasing the facet loads, which may be beneficial in relieving the pain of patients suffering from lumbar degeneration. Further, the less loading to surgical and adjacent facet may cause less degenerative changes to the facet joints.

The PSF system is associated with the limitation of motion at the fusion level and may cause excessive movement at the segments above and below the fusion level, and increases the degeneration of the adjacent segments [1, 27]. Conversely, a comparative biomechanical study showed that IPD reduces the ROM, load on a disc, and articular process stresses [30]. Therefore, IPDs can preserve a more normal anatomy of the spine and cause less violation to adjacent facet joints, resulting in a lesser probability of degeneration of the adjacent facet [31]. In our study, the TAU model showed less facet loads at adjacent level than PSF, and demonstrated relatively lesser motion in the adjacent segments when compared to that of the PSF model. Furthermore, the IDP at the adjacent levels was not significantly increased by the IPD, which may be an advantage of IPD for preventing ASD.

However, previously established IPDs have certain weak points, which cannot effectively control the axial rotation and lateral bending. Wang et al. revealed that the SPIRE device provided considerable stability in flexion and extension; moreover, the limitation of motion was similar to the bilateral PSF system, but the SPIRE device demonstrated a less stabilizing effect in lateral bending and axial rotation [17]. In our study, the ROM of the TAU model combined with interbody cage in lateral bending and axial rotation at the surgical level was lower than that of the SPIRE model, and it could overcome the limitation of the previously established SPIRE device. In addition, SPIRE has shown certain complications, such as posterior migration of interbody cage to the spinal canal, in a clinical study [15]. The posterior migration of the interbody cage to the spinal canal is due to the excessive flexion motion at the surgical level. Therefore, the TAU device was designed to endure more loads and limit the motions during flexion movement. As demonstrated in the results, the remaining ROM during flexion at surgical level was smaller in the TAU model than the SPIRE model. As the TAU model stabilized the surgical level more than the SPIRE during flexion motions, it could reduce the posterior migration of the interbody cage. However, this mechanism is hypothetical and a further prospective clinical study will be required for validation.

**Fig 6:**
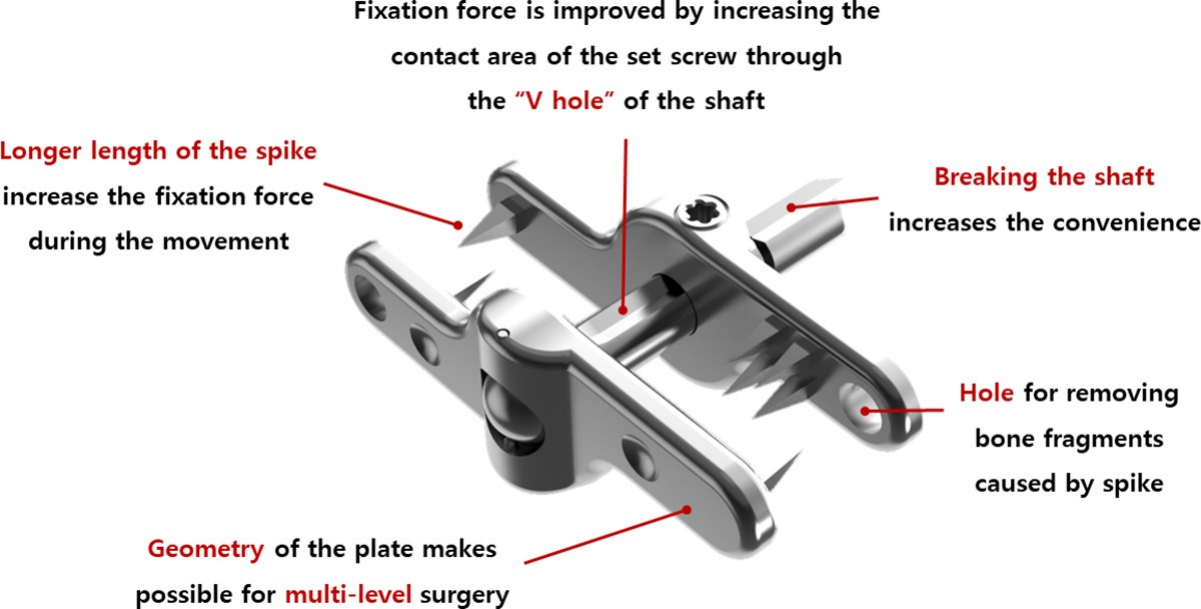
Features of the TAU device.

The newly developed device, TAU, is different from SPIRE in several aspects of its design (Fig 6). In addition, TAU has six spikes that are 4.5 mm in length, while SPIRE has eight spikes that are 3.2 mm in length. This longer length of the spike in the TAU device may increase the fixation force during the movement. Further, the fixation force is improved by increasing the contact area of the set screw through the “V hole” of the shaft. TAU consists of a ball-and-socket joint, where two plates and shafts meet; consequently, the TAU device and spinous process are conjoined at an appropriate angle with a larger force than that in the SPIRE, depending on the shape of the spinous process. Therefore, ROMs at the surgical level in all directions decreased considerably more in the TAU model than in the SPIRE model. Further, when conjoined with a posterior interbody cage, it can endure more loading at the surgical level during flexion movement and can reduce the retropulsion of the interbody cage to the spinal canal. While the SPIRE device has a straight shape, the TAU is shaped similar to a “Z,” which permits multilevel surgeries. In addition, the TAU device has a hole at the plate to remove bone fragments caused by spikes. Further, it is possible to break the shaft after locking the set screw in a TAU device; this may increase the convenience of the procedure. In addition, the convenient and easy characteristics of the TAU device may reduce the possibility of malposition during surgery, which was assumed to be a reason for postoperative complications [32, 33].

There are certain limitations of this study that must be acknowledged. One of the most important limitation is that the results of this study were obtained by finite element analysis and should be revealed through actual clinical studies in the future. Second, only one unique lumbar spine FE model was developed, so it may represent only a small fraction of human lumbar spines. Moreover, the components of the lumbar spine were simplified as linear elastic. Third, the present FE model did not include the characteristics of real spine such as degeneration, dehydration of disc, reduced disc height, hypertrophy of ligament, osteoporosis, and fracture of spinous process, there may be a difference from the actual clinical trial. Also, we did not consider the loosening of an implant and assumed that only complete fusion between bone and implant after surgery was achieved. The actual fusion effect between the bone and implant, duration of fusion, and failure of implants must be investigated through clinical studies. Therefore, such comparative studies and clinical trials should be conducted as future work. However, FE studies, unlike in vitro studies, can help researchers to examine the effects of the different devices on load sharing and stresses in the lumbar spine.

In summary, the FE analysis results show that the adjacent level effects of using the TAU device were superior to those using the PSF system. Furthermore, implanting the TAU device is simple and involves essentially no risk of nerve root injury and cerebrospinal fluid leakage because it is implanted on the spinous process. Further, the biomechanical results demonstrated that the TAU device provides fixation that is comparable in stability with PSF and with considerably less concomitant risk. Moreover, the TAU model stabilizes the surgical level more than SPIRE. When compared to other IPDs and the PSF, TAU demonstrated less compensation at adjacent levels in terms of ROM, IDP, disc stress, and facet loads, which may lower the incidence of ASD after fusion surgery. Consequently, the TAU device may be an attractive alternative to other systems. Thus, TAU can be used for the treatment of lumbar degenerative disease; moreover, it is expected to reduce the potential for degenerative changes at adjacent levels.

## Conclusion

In this study, we used a L1-S1 FE model to investigate the biomechanics of a newly developed IPD. This newly developed IPD, called TAU, was designed to stabilize the lumbar spine, not only to control lumbar spine movements, but also to minimize the loads on the adjacent level. Based on the simulated results, it exhibited similar stabilization effect at the surgical level and less biomechanical changes at the adjacent levels relative to PSF. The TAU device can be considered as an alternative system for treating degenerative lumbar disease while maintaining the physiological properties of the lumbar spine and minimizing the degeneration of adjacent segments.
